# A juvenile bird with possible crown-group affinities from a dinosaur-rich Cretaceous ecosystem in North America

**DOI:** 10.1186/s12862-024-02210-9

**Published:** 2024-02-09

**Authors:** Chase Doran Brownstein

**Affiliations:** 1 Department of Ecology and Evolutionary Biology, Yale University, New Haven, CT USA; 2 Stamford, USA

**Keywords:** Aves, Cretaceous, Crown Bird, Lance Formation, Paleontology

## Abstract

**Background:**

Living birds comprise the most speciose and anatomically diverse clade of flying vertebrates, but their poor early fossil record and the lack of resolution around the relationships of the major clades have greatly obscured extant avian origins.

**Results:**

Here, I describe a Late Cretaceous bird from North America based on a fragmentary skeleton that includes cranial material and portions of the forelimb, hindlimb, and foot and is identified as a juvenile based on bone surface texture. Several features unite this specimen with crown Aves, but its juvenile status precludes the recognition of a distinct taxon. The North American provenance of the specimen supports a cosmopolitan distribution of early crown birds, clashes with the hypothesized southern hemisphere origins of living birds, and demonstrates that crown birds and their closest relatives coexisted with non-avian dinosaurs that independently converged on avian skeletal anatomy, such as the alvarezsaurids and dromaeosaurids.

**Conclusions:**

By revealing the ecological and biogeographic context of Cretaceous birds within or near the crown clade, the Lance Formation specimen provides new insights into the contingent nature of crown avian survival through the Cretaceous-Paleogene mass extinction and the subsequent origins of living bird diversity.

**Supplementary Information:**

The online version contains supplementary material available at 10.1186/s12862-024-02210-9.

## Introduction

Birds form the largest radiation of flying vertebrates and include over ten thousand living species that display exceptional anatomical, behavioral, and ecological diversity. Although their dinosaur origins are now firmly established, the early evolution of the avian crown remains poorly known. This gap in the evolutionary history of living birds is nearly unparalleled among other major vertebrate crown clades [1–5] and is largely attributable to two factors. First, living birds have an exceptionally poor early fossil record. Few unambiguous crown birds are known from the Mesozoic [2, 6–9], and the fossil records of most major living avian lineages are limited to a handful of fossils or wholly nonexistent [10]. Second, analyses of both massive morphological [11] and genomic datasets have failed to produce a consilient picture of the interrelationships of major living bird clades, presenting one of the greatest challenges in phylogenetics [12–18].

Here, I use computed tomography to describe a probable Mesozoic crown bird based on an associated skeleton of a juvenile individual from the latest Cretaceous Lance Formation of Wyoming. The provenance of this specimen provides new evidence against the classically hypothesized southern hemisphere origins of living birds. The prehistoric ecosystem inhabited by the crown bird included numerous representatives of extinct stem-birds and bird-like dinosaurs ( [19]; this paper), providing evidence for the direct coexistence of non-avian theropods and the earliest representatives of the extant avian radiation.

## Results

### Systematic paleontology

Avialae Gauthier 1986.

Neornithes Gadow 1892.

?Neognathae Pycraft 1900.

?Galloanserae Sclater 1880.

?Galloanserae indet.

#### Description

**Fig. 1:**
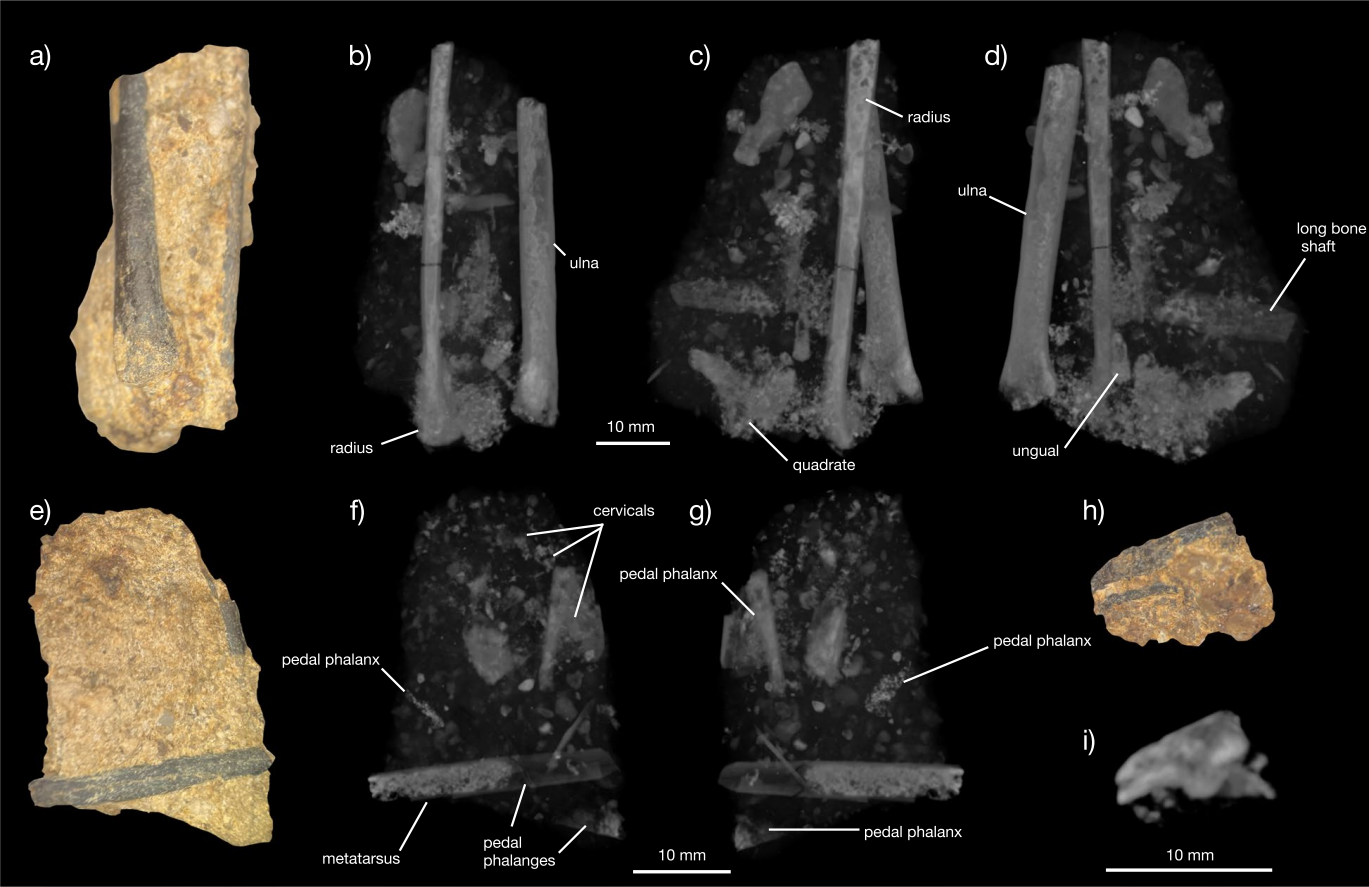
Preservation of YPM VP 59473. The blocks containing all bones of the holotype (except for the humerus, tibiotarsus, synsacrum fragment, and large distal pedal phalanx) are shown under light microscopy (**a**, **e**, **h**) and with multiple x-ray views of the largest (**b**, **c**, **d**), second largest (f, g), and smallest (**i**) blocks as rendered in VGStudio, showing the relative placement of bones in the matrix blocks

**Fig. 2:**
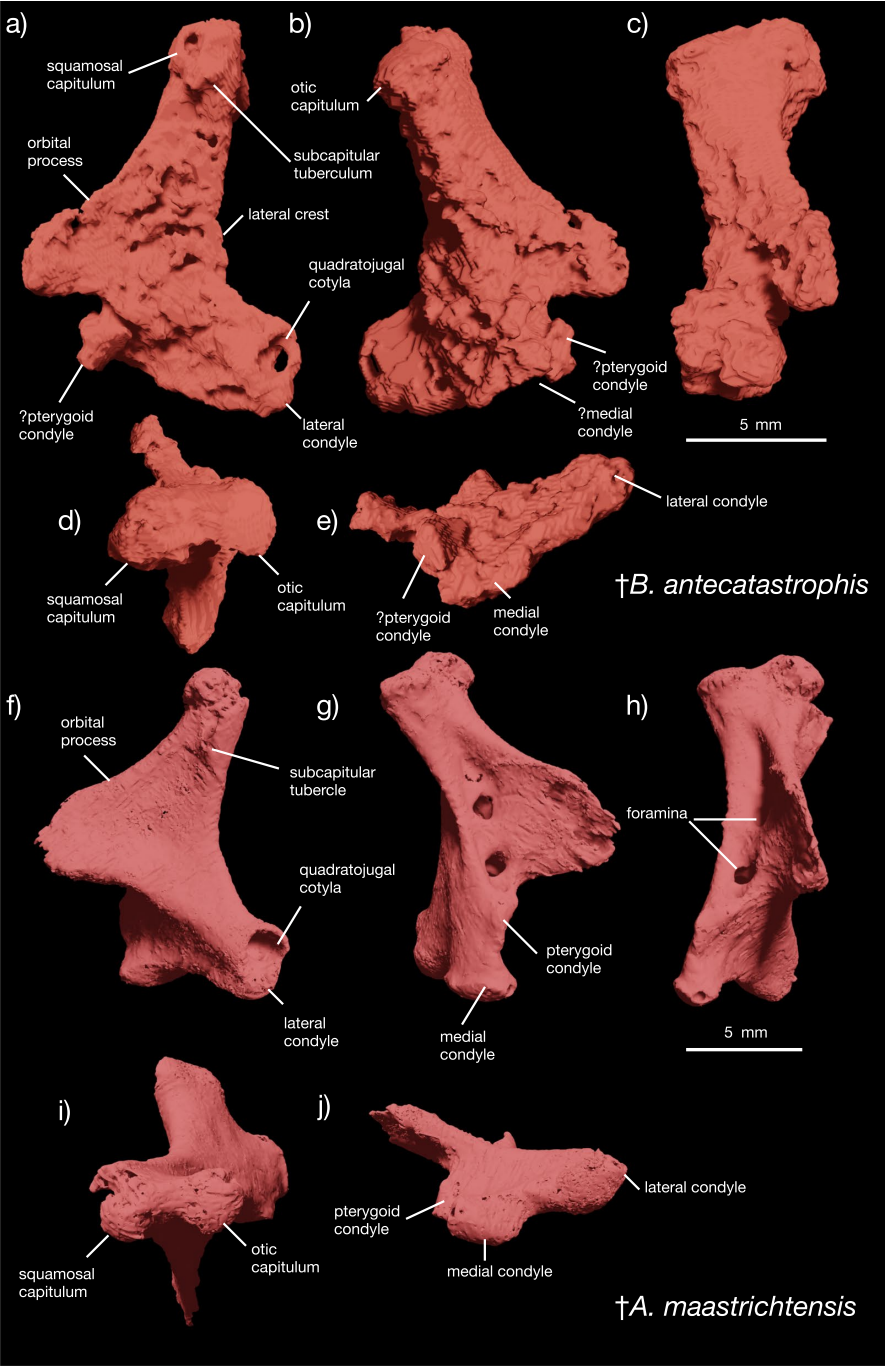
Quadrate of YPM VP 59473 and of †*Asteriornis maastrichtensis*. Quadrate of YPM VP 59473 in (**a**) lateral, (**b**) medial, (**c**) anterior, (**d**) dorsal, and (**e**) ventral views, compared to the quadrate of †*Asteriornis maastrichtensis* in (f) lateral, (**g**) medial, (**h**) anterior, (**i**) dorsal, and (**j**) ventral views

**Fig. 3:**
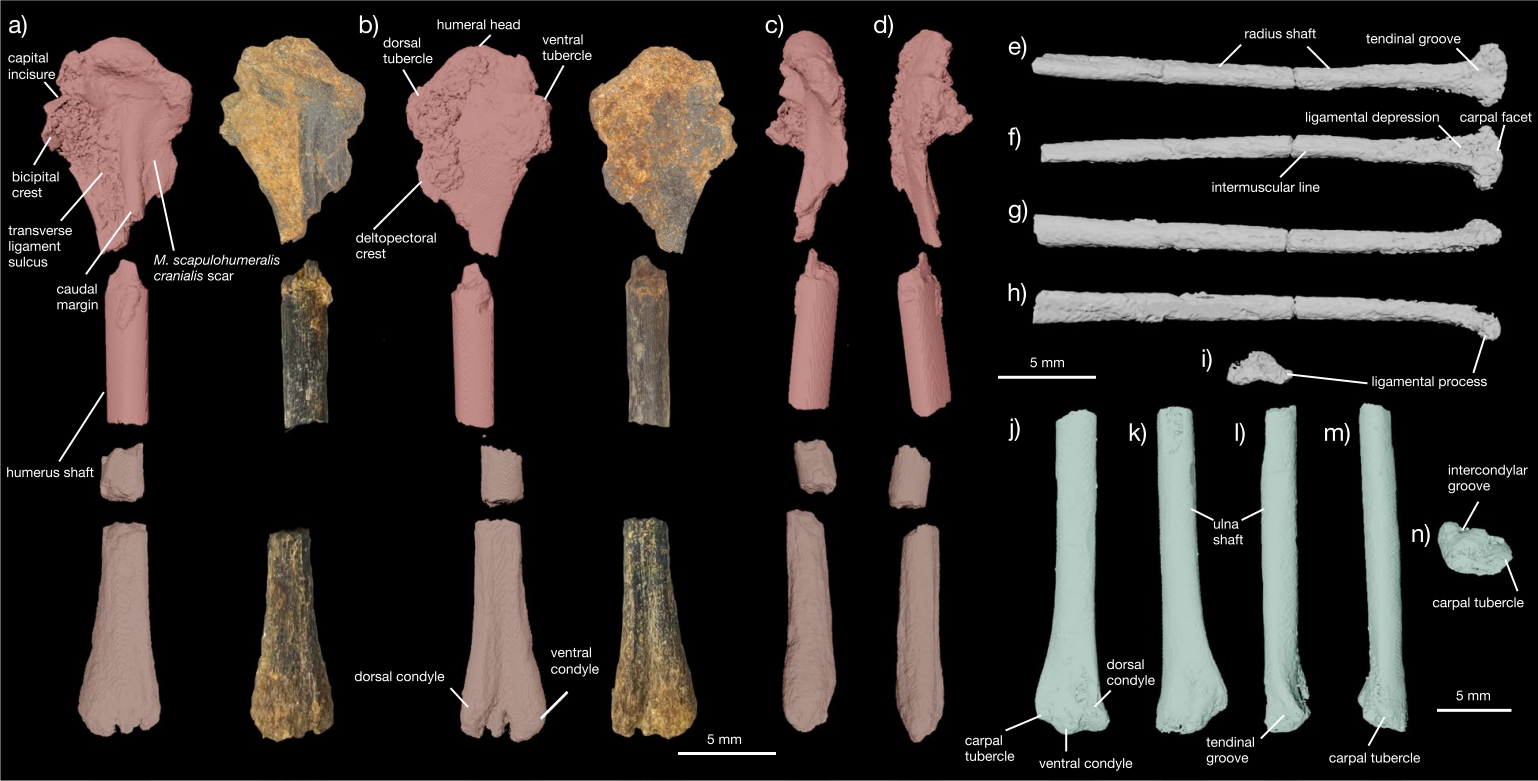
Forelimb of YPM VP 59473. Humerus in (**a**) posterior, (**b**) anterior, (**c**) lateral, and (**d**) medial views. In (**a**) and (**b**), both CT scans and color images are shown. Radius in (**e**) anterior, (**f**) posterior, (**g**) lateral, (**h**) medial, and (**i**) distal views. Ulna in (**j**) posterior, (**k**) anterior, (**l**) lateral, and (**m**) medial views

**Fig. 4:**
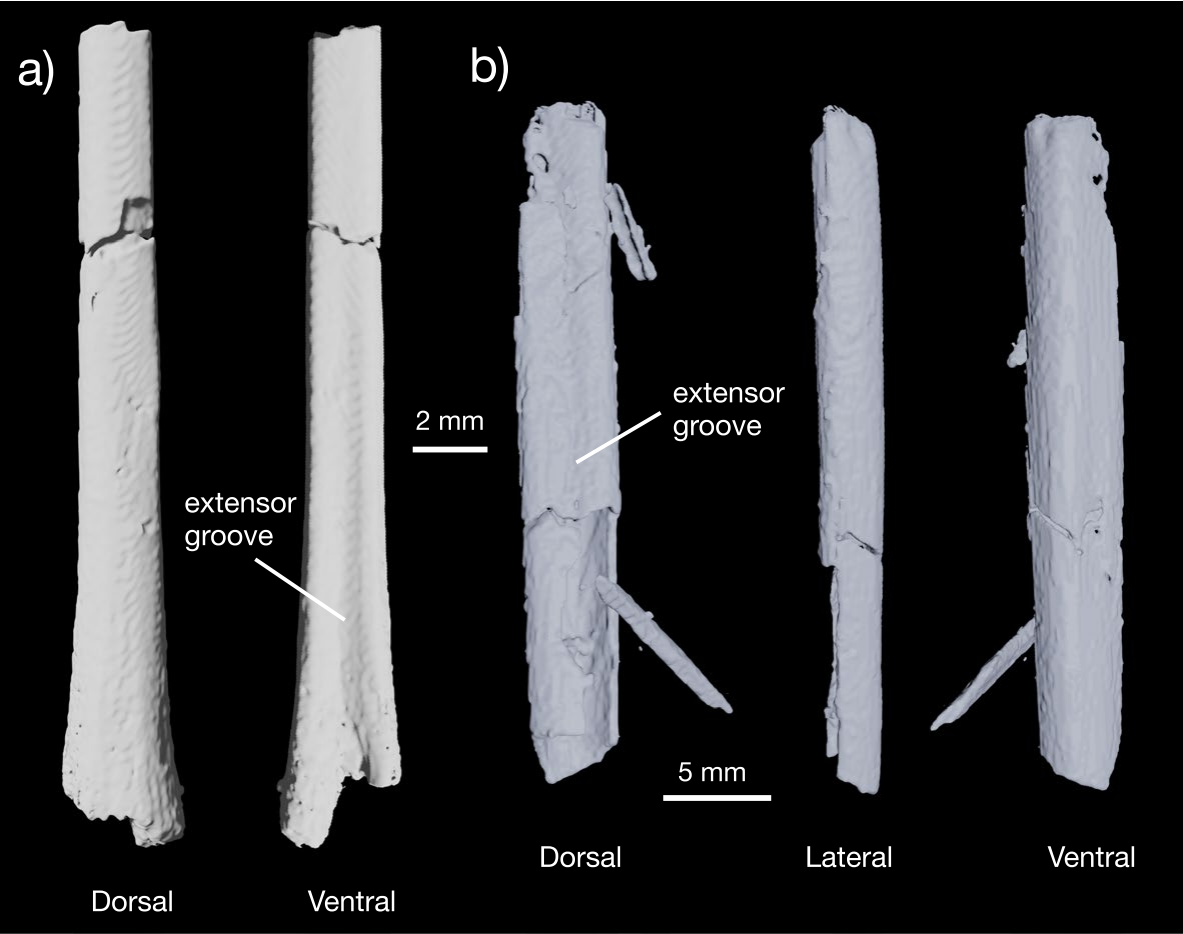
Hindlimb of YPM VP 59473. Tibiotarsus (**a**) and tarsometatarsus shaft (**b**) in multiple views

**Fig. 5:**
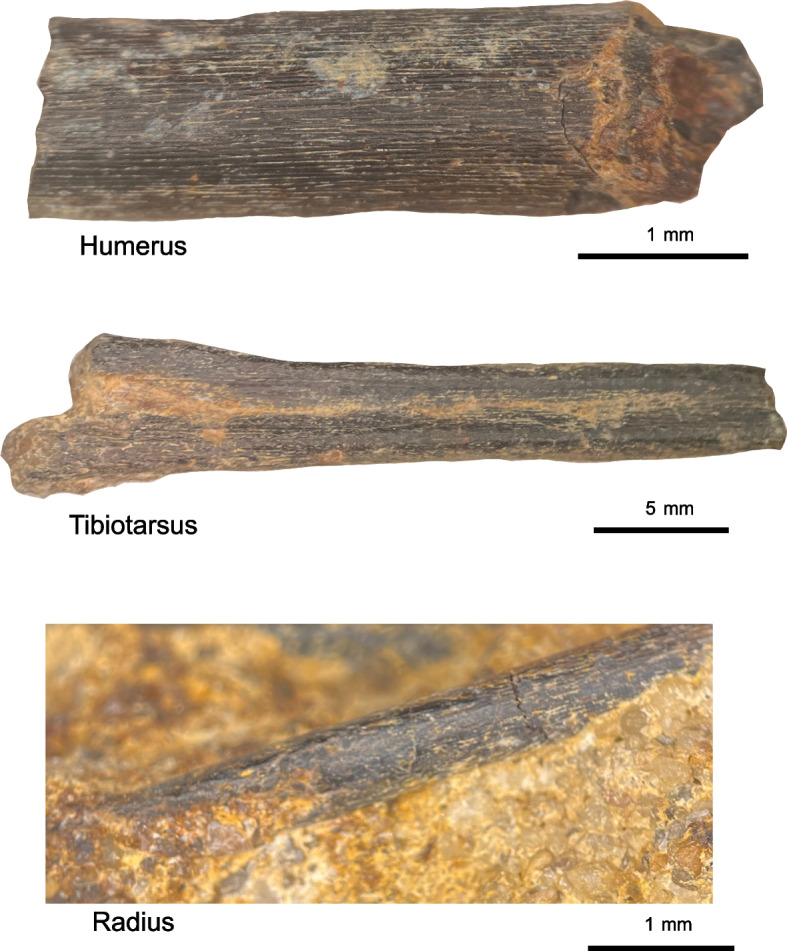
Ontogenetic status of YPM VP 59473*.* Light microscopy of freed and in situ bones shows clear striated patterning across unfinished bone surfaces identical to bones of ontogenetically immature birds from the Recent

Yale Peabody Museum Vertebrate Paleontology Collections (YPM VP) 59473, a partial skeleton consisting of the complete left quadrate, portions of the skull roof a partially articulated, though very poorly preserved, cervical series, a fragment of the synsacrum, the left humerus, the articulated left radius and ulna, partial left tibiotarsus, and partial pes. The specimen (Figs. 1, 2, 3, 4 and 5) is preserved in course-grained sediment that also included a small non-avian theropod tooth (identifiable based on the presence of serrations, and plausibly assignable to Tyrannosauroidea or Dromaeosauridae; Supplementary Text III). The bones are all closely associated or articulated (radius and ulna) in three small (< 5 cm) long blocks of conglomerate. Because no bones overlap and all show the same ontogenetic indicators (striated bone texture), they most likely come from a single individual. All bones except for the humerus, tibiotarsus, and a large partial pedal phalanx are embedded in matrix. Matrix portions also still cling to spots on these prepped-out bones. The quadrate was embedded in what appears to be an iron or bone-flake rich region of sediment in the block, and so its external surfaces appear less well preserved on the CT scan render than other bones.

#### Locality and horizon

Niobrara County, Wyoming, United States, North America. The fossil was collected from the Lance Formation by the J.B. Hatcher expedition, 1890–1904. The Lance Formation is a Maastrichtian-age unit that crops out across the basin and range region of the western U.S. Previously described avian fossils from the YPM collections recovered from the Hatcher expedition to Niobrara County are assumed to come from the upper portion of the Lance Formation, making them up to 0.65 million years older than the Cretaceous-Paleogene boundary ( [19]). Consequently, the age of YPM VP 59473 can be constrained to between 66.02 and 67.0 million years ago.

#### Referral

Crown avian and galloanserine affinities for YPM VP 59473 are supported by: clear separation of the otic and squamosal capitula on the quadrate (Fig. 2d); presence of a subcapitular tuberculum below the squamosal capitulum on the quadrate (Fig. 2a); expansion of the ventral condyles and pterygoid condyle on the quadrate (Fig. 2a-c, e); humeral head dorsally offset from the rest of the proximal margin of the humerus (Fig. 3a, b); tricipital fossa is deeply excavated (Fig. 3a, b); dorsal tubercle of the humerus is large and offset from the rest of the proximal margin (Fig. 3a, b).

#### Ontogenetic assessment

Following the comprehensive review of osteological indicators of ontogenetic status presented in [20], I conducted a survey of YPM VP 59473 which revealed its juvenile status (Fig. 5). Features unambiguously indicating ontogenetic immaturity in birds present in this specimen include the presence of unfinished, heavily striated bone surfaces [20–22]. Histological sections were not made due to the fragmentary nature of the specimen. Among the characters used to assign YPM VP 59473 to crown Aves and to Galloanserae, several deserve comment because they appear to change during ontogeny in stem and crown birds: the shape of the humeral head, development of the dorsal tubercle, and the depth of the tricipital fossa. In both juvenile and adult stem birds (for example, †*Archaeorhynchus* and †*Ichthyornis*), these features are weakly developed [23–25]. In crown birds, the dorsal tubercle and the humeral head become progressively more prominent with ontogeny (for example, *Phalacrocorax capillatus*; Fig. 3A in [22]. Thus, even accounting for the juvenile status of the specimen, the morphology of YPM VP 59643 is inconsistent with the hypothesis that it is a juvenile stem bird.

## Description

### Primary firsthand comparative material

Yale Peabody Museum Vertebrate Paleontology Collections.—YPM VP 1450, 1724, 1732, 1733, 1741, 1755, 1775, †*Ichthyornis dispar*, YPM VP 1734, †*Iaceornis marshi,* YPM VP 1200, 1472, 1476, 1477, †*Hesperornis regalis*, YPM VPPU 17324, †*Avisaurus archibaldi*, YPM VP 830, 835, †*Paleotringa* spp., YPM VP 850, 855, †*Graculavis* spp., YPM 147, †*Gastornis gigantea*, YPM VP 845, †*Telmatornis affinis*, YPM VP 948, †*Anatalavis rex*, YPM 102518, *Crypturellus noctivagus,* YPM 102520, *Casuarius casuarius*, YPM 15906, *Branta canadensis*, YPM 84420, *Anas platyrhynchos*, YPM 137647, *Leipoa ocellata*, YPM 102549, *Grus grus*, YPM 102272, *Musophaga rossae*, YPM 149811, *Opisthocomus hoazin*, YPM 84809, *Cariama cristata*, YPM 110763, YPM 110766, YPM 102334, *Nestor notabilis*, YPM 102276 *Sagittarius sagittarius*, YPM 104786, *Corvus novaezealandiae*, YPM 110024, *Phaethon rubricauda*, YPM 102125, *Fulmarus glacialis.*

French National Museum of Natural History.—MNHN AC 7994, †*Ludiortyx hoffmani*.

Mongolian Geological Institute—IGM 100/1017, †*Apsaravis ukhaana.*

#### Description

YPM VP 59473 is from a small crown bird; size estimation using the regression equation for humerus length in [26] and an estimated length of 70.0 mm for the nearly complete humerus of YPM VP 59473 gives a value of 413.4 g, which is close to the mass of the †*Asteriornis maastrichtensis* holotype individual estimated using the same dataset [2].

##### Quadrate

The left quadrate (Fig. 2a-e) is completely preserved, though slightly abraded along the lateral surface of its main body. The quadrate is similar to those of the early galloanserines †*Asteriornis maastrichtensis* (Fig. 2f-j) [2], †*Presbyornis pervetus* [27, 28], and †*Gallinuloides wyomingensis* [29], but differs considerably from the quadrates of early anseriforms like †*Conflicto antarcticus* [2, 30] or crown anseriforms and galliforms [2, 28] such as *Branta canadensis*, *Anas platyrhynchos*, and *Leipoa ocellata*. A key apomorphy of Neognathae, the distinct separation of the otic and squamosal capitula at the dorsal end of the quadrate [2], is present in the quadrate of YPM VP 59473 (Fig. 2d). In stem-group ornithurans like †*Ichthyornis dispar* and †*Apsaravis ukhaana*, the capitula are not delimited by a deep intercapitular sulcus ([83]; [31]), and in Paleognathae, these form a single proximal process rather than two distinct apices ( [32]). This lack of separation is present in specimens of †*Ichthyornis dispar* that are of different sizes [31]. Unlike the Lance Formation bird quadrate UCMP 53969 ( [28]), which possess an otic capitulum that is larger and more circular than the squamosal capitulum, the otic and squamosal capitula in the quadrate of YPM VP 59473 are of approximately equal size and shape (Fig. 2c, d). Similarly shaped capitula of equal size are also found in †*A. maastrichtensis* (Fig. 2h, i).

The main body of the quadrate in YPM VP 59473 is dorsoventrally straight and pillar-like, with slightly concave anterior and posterior margins. Unlike †*Asteriornis maastrichtensis* (Fig. 2g, h), †*Presbyornis pervetus* [28], †*Conflicto antarcticus* [2, 30], the Lance ‘galloanserine’ quadrate described by [9], gastornithiforms [31–33], and crown galliforms [2, 27, 28], but similar to most crown anseriforms [2, 28], the quadrate of YPM VP 59473 lacks any identifiable large foramina on the surface of its main body surrounding the orbital process (Fig. 2b, c). A single foramen, the basiorbital foramen, is present in ornithurans close to the crown [34], suggesting that the absence of these in YPM VP 59473 is an autapomorphy. However, the phylogenetic polarity of quadrate main body foramina is unclear. Further, the juvenile status of YPM VP 59473 may imply that main body foramina were present but very small due to ontogeny, as may be the case for one of the foramina in †*Asteriornis maastrichtensis* [2]*.* Further, damage to the surface of the quadrate in YPM VP 59473 might obscure the presence of foramina. The orbital process is developed into a triangular flange that expands from slightly over half of the dorsoventral axis of the quadrate main body in YPM VP 59473 (Fig. 2a-e), as in Ornithurae [2, 28, 34, 35]. Based on the preserved bone surface, the orbital process is slightly shorter than in †*Ichthyornis dispar* [34] and †*Conflicto antarcticus*, but similar to the process in †*Asteriornis maastrichtensis* (Fig. 2f, g), Galliformes, and *Anseranas semipalmata* [2]. However, the apex of the orbital process tends to be broken off in fossil quadrates, so the extent of this feature in YPM VP 59473 should be treated as uncertain.

The ventral end of the quadrate is developed into three major features: the lateral and medial condyles and what appear to be the base of the pterygoid condyle. The quadratojugal cotyla is positioned just dorsal to the lateral condyle, and consists of a deep, circular socket with a complete rim (Fig. 2a). The rim of the quadratojugal cotyla is socketed in crown galliforms and some crown anseriforms, but invariably lacks a notch in pan-anseriforms like †*Presbyornis pervetus* [2, 27, 28] and †*Conflicto antarcticus* [2, 30], as well as the pan-galloanserine †*Asteriornis maastrichtensis* [2] and the Lance ‘galloanserine’ quadrate UCMP 53969 [9].

##### Humerus

The humerus (Fig. 3a-d) of YPM VP 59473 is much better preserved than the humeri of the galloanserine †*Asteriornis maastrichtensis* [2] and allows for comparison with the complete humeri of the putative Mesozoic stem and crown neornithines †*Vegavis iaii* [6–8, 36]*,* †*Antarcticavis capelambensis* [37], †*Maaqwi cascadensis* [38], †*Telmatornis affinis*, and †*Tingmiatornis arctica* [39]. The proximal end of the humerus YPM VP 59473 is marked by its large, prominent, and strongly convex globose humeral head and less developed dorsal and ventral tubercles (Fig. 3a, b), which compares favorably with crown neornithines, †*Vegavis iaii* [6–8, 36], and †*Antarcticavis capelambensis* [37], but differs from the condition in ichthyornithines [23, 24, 40], †*Tingmiatornis arctica* [39]*,* and stemward birds, in which humeral head is variously globose but is not strongly offset from the rest of the proximal margin of the humerus. 

The dorsal tubercle of the humerus in YPM VP 59473 is strongly developed and offset from the humeral head. The dorsal tubercle is approximately as mediolaterally wide as anteroposteriorly long (Fig. 3a, b). These neornithine synapomorphies distinguish YPM VP 59473,  †*Vegavis iaii* [6–8, 36], and †*Antarcticavis capelambensis* [37] from non-neornithine ornithurans, such as ichthyornithines [23, 24, 40] and †*Apsaravis ukhaana* [35]. The ventral tubercle is present and prominent, but markedly smaller than in †*Ichthyornis dispar* [23, 24] or †*Janavis finalidens* [40]. The development of the ventral tubercle appears to vary both intraspecifically and ontogenetically in ichthyornithines [23, 24, 40]. The tricipital fossa is deeply concave and extensive, spanning the medial surface of the proximal humerus between the margin of the bicipital crest and the caudal margin (Fig. 3a, b) as in living neornithines, †*Vegavis iaii* [6–8] and †*Antarcticavis capelambensis* [37]*,* but unlike †*Ichthyornis dispar* [23, 24], †*Janavis finalidens* [40], †*Apsaravis ukhaana* [35], or †*Tingmiatornis arctica* [39].

The bicipital crest of the humerus of YPM VP 59473 is large (Fig. 3a-d). This crest is marked on its dorsal surface by the transverse ligament sulcus, which is deep, clearly marked, and linear as in neornithines but unlike the condition in †*Ichthyornis* *dispar* [23, 24], †*Janavis finalidens* [40]*,* or †*Apsaravis ukhaana* [35], where the sulcus is shallow and rounded. The bicipital crest sits opposite to the deltopectoral crest, which is incompletely preserved. The deltopectoral crest is less distally extensive (measures less than 25% of the long axis of the humerus) than those of †*I. dispar* [23, 24], †*J. finalidens* [40], †*A. ukhaana* [35], or †*Tingmiatornis arctica* [39], but compares favorably with †*Vegavis iaii* [6–8]*,* †*Antarcticavis capelambensis* [37], and early-diverging crown neornithines [10, 36, 41]. The caudal margin is also well developed in YPM VP 59473 relative to ichthyornithines and other stem birds close to the crown. †*T. arcticus* also possesses a strongly developed caudal margin of the humerus, despite sharing numerous features with ichthyornithines and not neornithines. The polarity of this character state remains unresolved even though neornithines appear to invariably show prominent caudal humerus margins. The distal end of the humerus of YPM VP 59473 is poorly preserved but does appear to show equally developed dorsal and ventral distal humeral condyles based on the shape and size of their medial portions (Fig. 3a-d).

##### Radius

The radius (Fig. 3e-h) was preserved in articulation with the ulna on the surface of the larger of the two blocks included in the holotype of YPM VP 59473 (Fig. 1). The radius is very long and slender, with a straightened shaft (Fig. 3e-h). The surface of the radial shaft is heavily striated and lacks a finished surface, a feature indicative of the juvenile ontogenetic status of the holotype specimen of YPM VP 59473; this feature is also clearly present on the humerus, ulna, and distal tibiotarsus, although the radius is the best example of this texture on a bone still laying in situ. However, the medial surface of the radius, which is still encased in matrix, shows a conspicuous intermuscular line running down the shaft surface. This intermuscular line is found in living Aves and †*Ichthyornis dispar* [23, 24].

The anterior surface of the distal end of the radius is marked by a large, round, and shallow sulcus identified as the tendinal groove. This feature lacks the prominent associated ridge found in extinct ornithurans like †*Ichthyornis dispar* [23] and is generally comparable to the weak tendinal groove found in neornithines (e.g., †*Conflicto antarcticus*, [30]). Posteriorly, there is a shallow ligamental depression and a weakly developed carpal facet. As in *Anseranas semipalmata* [41], but unlike stem [29, 42] and crown galliforms (pers. obs.), some anatids [41] (pers. obs. of *Branta canadensis*), †*Ichthyornis dispar* [23], or †*Apsaravis ukhaana* [35], the distal end of the radius is heavily expanded dorsoventrally. The ligamental process is short.

##### Ulna

The ulna (Fig. 3j-n) was preserved in articulation with the mostly complete radius on the surface of the larger of the two blocks included in the holotype of YPM VP 59473 (Fig. 1). The partial ulna, which includes at least one third of the shaft and the complete distal end, is uncrushed. The shaft is straight along the long axis of the ulna, curving only at its distal end to meet the radius at the wrist. The distal end of the ulna is approximately twice as wide mediolaterally as anteroposteriorly and has three major processes: the dorsal and ventral distal ulnar condyles and the carpal tubercle. Unlike †*Ichthyornis dispar* and stem birds within or close to Ornithurae [23, 24, 35], but similar to many neornithines (pers. obs., see specimens noted above), the carpal tubercle of the ulna is poorly developed and only differentiated from the rest of the bone by a shallow groove. The ovoid dorsal and ventral condyles are poorly developed and separated by a shallow intercondylar groove. Laterally, the surface of the dorsal condyle is marked by a shallow tendinal groove that runs proximodistally along the surface of the distal ulna.

##### Tibiotarsus

Some of the shaft and the eroded distal end of the left tibiotarsus were preserved (Fig. 4a). This bone is elongated and more gracile than the tibiotarsi of †*Ichthyornis dispar* [23, 24] or †*Apsaravis ukhaana* [35], but similar to †*Vegavis iaii* [7], †*Polarornis gregorii * [8]*,* and early neognaths [2, 10, 29, 41, 42]. A shallow extensor groove runs across the anterior surface of the bone. Generally, the morphology of the tibiotarsus and tarsometatarsus (see below) indicates that the hindlimb of YPM VP 59473 was gracile and long.

##### Tarsometatarsus

The shaft of the tarsometatarsus is preserved on the surface of the smaller block included in the holotype of YPM VP 59473 (Figs. 1, 4 and 5) and is identifiable as such based on its flattened, elliptical shaft, shallow extensor groove, and discrete lateral and medial margins (Fig. 4b). Little can be said about the morphology of this bone except that the distal end of the metatarsus appears to be more elongated than in †*Ichthyornis dispar* [23, 24], hesperornithines (e.g., [43]), or †*Apsaravis ukhaana* [35], but similar to the Maastrichtian galloanserine †*Asteriornis maastrichtensis* [2]*,* and the possibly anseriform presbyornithids [41, 44, 45]. The possible Maastrichtian Antarctic neornithines †*Vegavis iaii* and †*Antarcticavis capelambensis* also all possess similarly elongated, fused tarsometatarsi such that the shaft of the bone shows little indication of each of the co-ossified bones [6–8, 37].

##### Pes

**Fig. 6:**
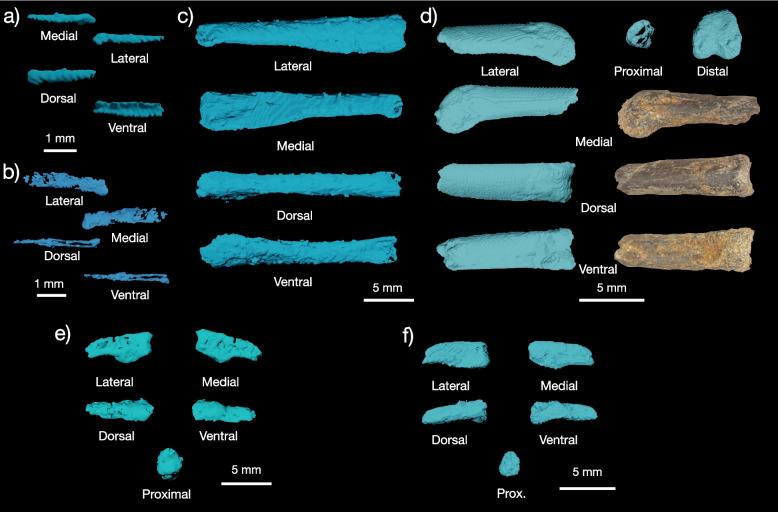
Pedal elements of YPM VP 59473. Pedal phalanges I-1? (**a**), I-1?(**b**), II-1? (**c**), III-1? (**d**), II-3? (**e**), and IV-5? (**f**) in multiple views. In (**d**), phalanx is shown in both color illustrations and CT scans

Six pedal phalanges and three possible pedal elements are preserved in the holotype of YPM VP 59473 (Fig. 1) with varying degrees of completeness (Fig. 6). Based on the morphology of the preserved phalanges, I reconstruct the pes of YPM VP 59473 as elongated, with pedal phalanges that decrease in length towards the distal ends of each digit (this is the condition in most other birds, with larger phalanges proximally). In this way, the pes of YPM VP 59473 compares well with †*Ichthyornis dispar* [23, 24], †*Janavis finalidens* [40], †*Vegavis iaii* [6–8], modern anseriforms like *Anas* and *Branta* (pers. obs.), and wading birds in the clade Neoaves (i.e., Gruiformes, Pelicaniformes; Charadriiformes; Mirandornithes), but contrasts markedly with the pes of stem and crown group galliforms [29, 42, 46], the ornithuran †*Apsaravis ukhaana* [35], and many other predominately terrestrial birds. The pes of YPM VP 59473 also differs from the specialized pes of penguins (Sphenisciformes; pers. obs.) and members of the clade †Hesperornithes, which are robust and asymmetrical [43]. Here, I focus on describing the six definite phalanges.

Two small, poorly preserved phalanges (Fig. 6a, b) are columnar and hollow internally, with slightly asymmetrical distal condyles separated by a deep intercondylar sulcus (Fig. 6a, b). However, the identification of these bones as phalanges is tentative, and they may be warped, fragmented portions of other long bones. One very elongated, slightly mediolaterally asymmetrical proximal phalanx (Fig. 6c) and the complete distal ungual (Fig. 6e) appear to represent either digits II or IV given their asymmetry. The ungual possesses a concave, elliptical proximal articular surface and a large, proximally positioned flexor tubercle on its ventral surface. The ungual body is weakly recurved. On the lateral and medial surfaces of the main body of the ungual, deep neurovascular grooves curve along the main axis.

Pedal digit III as reconstructed is represented by the distal third of the first phalanx, the proximal two-thirds of the second phalanx, and the complete third phalanx. These phalanges are bilaterally symmetrical and mediolaterally widened relative to the other bones of the pes. An asymmetrical distal end of the proximal phalanx (Fig. 6d) and a complete, poorly recurved, and slightly mediolaterally asymmetrical pedal ungual (Fig. 6f) are also likely from digits II or IV.

## Discussion

YPM VP 59473 sheds light on the biogeography of early crown birds and their closest relatives. Although stem birds are plentiful in the Late Cretaceous of North and South America [4, 5, 43, 47, 48], the record of Mesozoic crown birds from North and South America is restricted to a number of isolated bones of unclear phylogenetic position and provenance [4, 9].

The discovery of YPM VP 59473 provides additional evidence that bird species very near to and within the crown clade acquired a cosmopolitan distribution by the Late Cretaceous, which is logical given the inferred capability for flight in YPM VP 59473 and other derived ornithurans like †*Ichthyornis dispar* and †*Vegavis iaii* [3, 6, 7, 23]. On a larger scale, the provenance of YPM VP 59473 underscores the ambiguity surrounding the geographic origins of living bird diversity. The earliest-diverging clades of living birds, such as the paleognaths, megapodes, magpie goose *Anseranas*, and anhimids, are all found in the southern hemisphere, a pattern that appears to support to an origin for crown birds in the southern continents [49]. However, growing evidence from the fossil record has shown that these now exclusively southern hemisphere bird clades were once also distributed across the northern continents [10, 19, 50–53]; the current distributions of clades as varied as mousebirds, seriemas, and hoatzins appear to represent contractions of far wider prehistoric ranges [54] The presence of YPM VP 59473 in North America supports a modified view of early crown avian biogeography that featured early cosmopolitanism followed by recent radiations into and throughout the southern hemisphere.

**Fig. 7:**
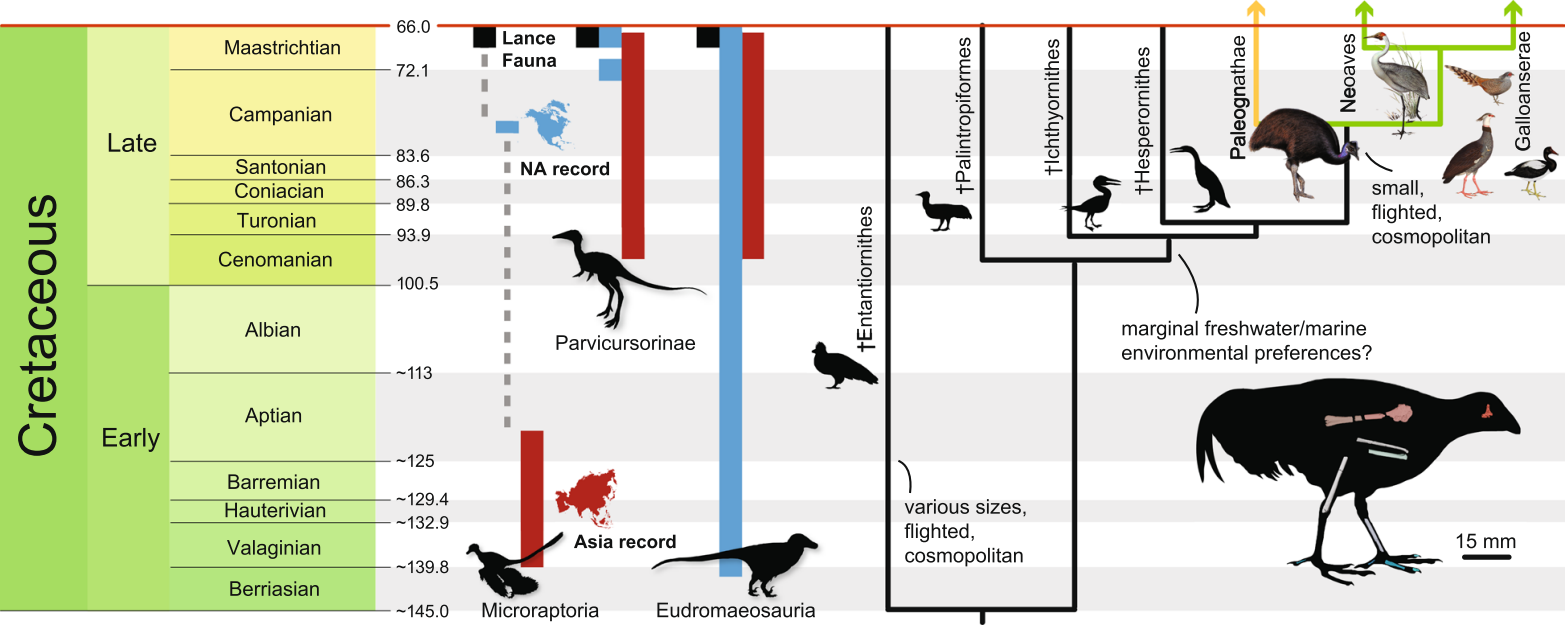
The ecological and temporal origins of living birds. Left side of the diagram shows the temporal and spatial range extensions and records of key small-bodied non-avian theropod clades found in the Lance Formation assemblage, and cladogram at right shows the major clades of stem and crown birds that survive to or past the K-Pg extinction, with ecologically relevant features that have been considered important to differential avian survival through that event noted along branches. All clades shown on tree are unambiguously represented in the Lance Formation assemblage, except Neoaves and Paleognathae. Divergence times for major lineages are primarily based on [2, 13, 14]. Outlines are public domain from phylopic.org or by the author. Bird illustrations are public domain by John Gould

The discovery of YPM VP 59473 also provides new information on the ecological context of early crown bird evolution and survival through the Cretaceous-Paleogene (K-Pg) mass extinction, which killed all non-avian dinosaurs and all major stem bird clades. The ecologies of living birds and early members of the crown indicate that the K-Pg extinction globally selected against arboreal and large-bodied species [3, 14, 55]. The Lance Formation ecosystem that YPM VP 59473 inhabited included a diverse assemblage of toothed stem-birds from at least four major clades (Fig. 7; [4]). A survey of the Lance Formation theropod fauna in the Yale Peabody Museum collections also shows that this unit has produced material assignable to eudromaeosaurian, alvarezsaurid, troodontid, and potentially ‘four-winged’ microraptorine dinosaurs (Supplementary Information). A serrated tooth resembling those of dromaeosaurids and small tyrannosaurids (e.g., [56, 57]) is included in YPM VP 59473 and apparently came from the same block of conglomerate. Although the formation that produced European crown bird †*Asteriornis maastrichtensis* has also yielded a handful of non-avian dinosaur fossils, these are isolated and fragmentary ‘bloat and float’ bones that were washed out to sea and scavenged [58], and therefore do not necessarily come from the same ecosystem. The preservation of a well-preserved theropod tooth in the same block as YPM VP 59473, as well as the diversity of small-bodied non-avian theropod material recovered from various horizons in the Lance Formation, provides strong evidence for the coexistence of crown birds and non-avian dinosaurs in the northern hemisphere.

The coexistence of YPM VP 59473 and a diverse fauna of bird-like theropods and stem-birds (Fig. 7) raises the question of what factors might have mediated crown bird survival across the Cretaceous-Paleogene boundary, particularly as many features thought to have aided the survival of crown birds, such as a littoral ecology [2, 44], arboreality and flight capacity [3, 59–61], dietary diversity [62–65], and terrestriality [3] are now known to have occurred across most of the major stem clades, including enantiornithines and ichthyornithines, that existed up to the time of the extinction. In sum, the anatomical and ecological information provided by YPM VP 59473 suggests that the survival of all three main clades of crown birds across the boundary (Fig. 7) is an excellent example of contingency in evolution. Rather than possessing a unique set of traits that facilitated their survival, crown birds may have been the ‘happy few’ that persisted across the K-Pg boundary as the more speciose and ecologically diverse stem-birds and non-avian dinosaurs went extinct around them.

## Methods

### Microscopy and measurements

Detailed characterization of the anatomy of bones and bone surfaces free of matrix was carried out using a standard light microscope at the Yale Peabody Museum. Ontogenetically informative characteristics were identified following several papers, especially [20–22] as well as via comparisons with juvenile bird skeletons at the YPM. Measurements of the specimen were taken using digital calipers and are included in Table S1.

### Computed tomography scanning and segmentation

Computed tomography (CT) scanning has facilitated the examination of three-dimensionally preserved Cretaceous avian fossils in exceptional detail without disturbing them from the matrix [2, 34, 40, 55]. The possibility that the conglomerate blocks included in YPM VP 59473 might store extensive avian material was considered following the description of †*Asteriornis maastrichtensis* and †*Janavis finalidens* from the late Maastrichtian of Belgium; both of these were recovered from block specimens [2, 40]. High-resolution CT scanning of YPM VP 59473 was conducted by M. Fox at the Yale Peabody Museum CT scanning facility. Scanning parameters are in Table S3. Individual elements were visualized and segmented using the programs 3D Slicer [66] and VGStudio MAX 3.5. Final renders were produced in the program Blender v 3.4.0.

### Lance formation faunal survey

The collections of the Yale Peabody Museum contain numerous small vertebrate fossils from the 1903 J.B. Hatcher expedition to Niobrara County, Wyoming, and subsequent collection trips to Lance Formation outcrop. To understand the ecological community of YPM VP 59473, I conducted a survey of all catalogued fossil avian and non-avian theropod material in the Yale Peabody Museum, with Supplemental survey information from previous publications (e.g., [4]). Comparative anatomical work was conducted using anatomical data collected from the Yale Peabody Museum, American Museum of Natural History, Muséum National D'histoire Naturelle, Denver Museum of Nature and Science, and New Jersey State Museum. Pie charts and other visualizations were made using the R 4.0.2 program ggplot2 3.4.2. The Supplementary Text contains additional description and assignment justifications for theropod fossils that are newly described in this contribution, which include the first probable records of microraptorines and alvarezsaurids of the genus †*Trierarchuncus prairiensis* [67] from the Lance Formation.

### Supplementary Information

**Additional file 1.**

**Additional file 2.**

**Additional file 3.**

**Additional file 4.**

## Data Availability

All data is available in the main text and supplementary files, and the raw CT data are uploaded to Morphosource at: https://www.morphosource.org/concern/media/000590271?locale=en, https://www.morphosource.org/concern/media/000590262?locale=en.
