# Correction: A juvenile bird with possible crown-group affinities from a dinosaur-rich Cretaceous ecosystem in North America

**DOI:** 10.1186/s12862-024-02216-3

**Published:** 2024-03-14

**Authors:** Chase Doran Brownstein

**Affiliations:** 1 Department of Ecology and Evolutionary Biology, Yale University, New Haven, CT USA; 2 Stamford, USA

**Correction: BMC Ecology and Evolution (2024) 24:20**

10.1186/s12862-024-02210-9

After publication of the article, it was brought to our attention that ‘†Borealornis antecatastrophis’ in paragraph 3 of the Humerus section should be changed to “YPM VP 59473”. Note that †*Borealornis* antecatastrophis is rendered a nomen nudum.

The original publication has been corrected.

