# Supplementary material for: A juvenile bird with possible crown-group affinities from a dinosaur-rich Cretaceous ecosystem in North America
Source: BMC Ecol Evol. 2024 Feb 9;24:20. doi: 10.1186/s12862-024-02210-9 (PMC10858573; doi:10.1186/s12862-024-02210-9)
Supplement: Supplementary file 1 — Additional file 1. [file 12862_2024_2210_MOESM1_ESM.docx]

**Supplement to: First Mesozoic crown bird from the Americas reveals the ecological context of avian survival through the end-Cretaceous extinction**

Chase Doran Brownstein^1, 2^*

^1^Department of Ecology and Evolutionary Biology, Yale University, New Haven, Connecticut

^2^Stamford Museum and Nature Center, Stamford, Connecticut

*Corresponding author, chase.brownstein@yale.edu

1. **Supplementary faunal survey results.**
2. **Supplementary References.**

**I. Supplementary faunal survey results.**

Theropoda Marsh 1881

Maniraptora Gauthier 1986

†Troodontidae Gilmore 1924

†Troodontinae indet.

*Material.—*YPM VP 004691, complete tooth crown attached to root (Figure S1a, b).

*Locality and Horizon.—*Niobrara County, Wyoming, United States, North America. The fossil was collected from the Lance Formation by the J.B. Hatcher expedition, 1890-1904.

*Comments.—*The crown and root (Figure S1a, b) is identifiable as the tooth of a troodontid based on a combination of features, including its slightly recurved main body, the presence of much larger distal than mesial denticles, and distal denticles that are large and strongly apically hooked (e.g., Currie, 1987; Larson and Currie, 2013; Williamson and Brusatte, 2014; Larson et al., 2016; Hendrickx et al., 2019). The specimen does not possess clearly developed mesial denticulation, which differs from the condition in † ‘*Troodon*’ but compares favorably to the teeth of troodontine troodontids from the Late Cretaceous of Asia, such as †*Saurornithoides mongoliensis* (Norell et al., 2009; Pei et al., 2017). YPM VP 004691 is not referable to the troodontid tooth taxon †*Paronychodon*, which lacks serrations and possesses large apicobasally-running striations, or †*Pectinodon*, which lacks strongly apically-hooked distal denticles and does not have a strongly recurved main body (Larson and Currie, 2013).

†Dromaeosauridae Matthew and Brown 1922

†Microraptoria Senter et al. 2004

Cf. †Microraptoria indet.

*Material.—*YPM VP 865 (in part), distal half of metatarsal III (Figure S1e-g), YPM VP 57403, ?left pedal ungual II-3 (Figure S1h-j), YPM VP 57404, ?left pedal phalanx II-1 (Figure S1k-o), YPM VP 57237, distal caudal vertebra (Figure S1p-r).

*Locality and Horizon.—*Niobrara County, Wyoming, United States, North America. The fossil was collected from the Lance Formation by the J.B. Hatcher expedition, 1890-1904. YPM VP 57237 was collected from Lance Quarry #1, and YPM 57403 was collected from Peterson’s Quarry.

*Comments.—*The distal half of metatarsal III YPM VP 865 is assignable to a non-arctometatarsalian theropod given that it is small, strongly dorsoventrally compressed, lacks a ventral median ridge delimiting the metatarsal IV and II buttressing surface, and does not present mediolateral thinning of the metatarsal shaft proximally; these conditions, together with the presence of articular surface for the other two metatarsals on either side of the metatarsal III shaft, match those in dromaeosaurids (Ostrom, 1969; Makovicky and Norell, 1998, Fowler et al., 2011). A dromaeosaurid affinity of the metatarsal is also indicated by a strongly ginglymoid distal articular surface (e.g., Turner et al., 2012). Although histological analysis could not be conducted on the specimen, the bone is inferred to be from a non-hatchling individual based on its smooth bone surface and well-ossified distal end (Griffin et al., 2020). The metatarsal III is tentatively referred to a microraptorine because of its small size, well-developed buttressing surfaces for metatarsals II and IV indicating close abutment of these bones, and elongation (e.g., Hwang et al., 2002; White, 2009; Pei et al., 2014; Lü and Brusatte, 2015; Poust et al., 2020). However, this bone might also belong to a small unenlagiine, as these dromaeosaurids show similar metatarsal features (e.g., Gianechini et al., 2018; Forster et al., 2020). Similarly, it may be from a halszkaraptorine (Turner et al., 2011; Cau et al., 2017; Cau and Madzia, 2018; Lee et al., 2022), although this clade is exclusively known from eastern Asia.

The left pedal ungual II-3 and phalanx II-1 are referred to †Dromaeosauridae and tentatively to Microraptoria following the justification of referring similar elements to that clade in Longrich and Currie (2009a). II-1 is elongated, with a spool-shaped articular surface and weakly dorsally expanded distal condyles compared to the strong expansion seen in †Eudromaeosauria (Longrich and Currie, 2009a) or the complete lack of expansion in †Halszkaraptorinae (Cau et al., 2017). The Lance phalanx II-1 is also less elongated than those of unenlagiines and has shallower collateral ligament pits at its distal end (e.g., Turner et al., 2012; Gianechini et al., 2018; Forster et al., 2020). The mediolaterally broad, recurved pedal ungual better resembles those of †*Rahonavis*, †*Buitreraptor*, and microraptorines than †Eudromaeosauria, which have asymmetrical vascular grooves and are much more strongly hooked (Longrich and Currie, 2009).

The distal caudal vertebra is the most diagnostic of the possible Lance microraptorine material. It shows a clear set of dromaeosaurid apomorphies, including anteroposteriorly elongation, the presence of a neural spine that is dorsally reduced to a thin ridge closely fused to the centrum, and a widened ventral fossa (e.g., Turner et al., 2012). Among dromaeosaurids, the extreme anteroposterior elongation observed in the Lance caudal centrum and the absence of extensive ossified ligament attachments along the vertebra, which is assumed to be from a non-hatchling individual based on fusion of the neural spine to the centrum and the smoothened external bone surface (Griffin et al., 2020), is strongly supportive of a microraptorine affinity (Hwang et al., 2002; Turner et al., 2012; Poust et al., 2020). Only the bases of the pre- and postzygapophyses are preserved, but these suggest that these features were rodlike as in other microraptorines (Hwang et al., 2002).

†Dromaeosauridae Matthew and Brown 1922

†Eudromaeosauria Longrich and Currie 2009

†Eudromaeosauria indet.

*Material.—*YPM VPPU 20589, proximal portion of pedal phalanx II-1 (Figure S1s).

*Locality and Horizon.—*Polecat Dome, Park County, Wyoming, United States, North America. The fossil was collected from the Lance Formation, mid-section 31, by Princeton University in 1947.

*Comments.—*The proximal pedal phalanx II-1 is of an adult theropod based on the presence of at least four lines of arrested growth in cross-section and the smooth external bone surface (Griffin et al., 2020). It is assignable to a eudromaeosaurian dromaeosaurid based on its heavily ventrally expanded, hatchet-shaped proximal surface, which is characteristic of that clade (Longrich and Currie, 2009), and belonged to a small-bodied dromaeosaurid less than 2 meters in length based on comparisons with the same element in other North American and Asian Late Cretaceous species (e.g., Sues, 1978; Makovicky and Norell, 1998; Longrich and Currie, 2009; Turner et al., 2021).

†Alvarezsauridae Bonaparte 1991

Cf. †*Trierarchuncus prairiensis* Fowler et al. 2020

*Material.—*YPM VP 56916, complete manual ungual I (Figure S2a-c), YPM VP 57236, complete pedal ungual (Figure S2d-f); YPM VP 57402, partial pedal ungual (Figure S2g-i).

*Locality and Horizon.—*Niobrara County, Wyoming, United States, North America. Recovered during the 1889 expedition lead by John Bell Hatcher.

*Comments.—*Recent work has conclusively demonstrated the presence of the small, bird-like family alvarezsauridae in North America (Hutchinson and Chiappe, 1998; Chiappe et al., 2002; Longrich and Currie, 2009b; Fowler et al., 2020; Friemuth and Wilson, 2021). Manual and pedal material of alvarezsaurids is highly diagnostic, even to the genus and species level, because of the specializations found in this lineage (Chiappe et al., 2002; Longrich and Currie, 2009b; Pittman et al., 2015; Averianov and Sues, 2017, 2022; Fowler et al., 2020; Friemuth and Wilson, 2021). The most complete specimen of an alvarezsaurid reported here is YPM VP 56916, the nearly complete manual ungual of digit I, which lacks only the distalmost extremity of the actual claw. The manual ungual is identified as that of a parvicursorine alvarezsaurid based on its robusticity, the presence of paired foramina on either side of the ventral surface of the proximal end, the reduction of the flexor tubercle to a low keel, and the equal mediolateral width and dorsoventral height of the proximal articular surface (Chiappe et al., 2002; Longrich and Currie, 2009b; Fowler et al., 2020; Friemuth and Wilson, 2021; Averianov and Sues, 2022). The ungual bears all the distinguishing features of the species †*Trierarchuncus prairiensis*, which until now has only been reported from the Hell Creek Formation (Fowler et al., 2020; Friemuth and Wilson, 2021). These are: the ventral sulcus extends proximally to the level of the distal termination of the paired foramina, but not past them; the paired foramina are deeply embayed laterally; presence of numerous spur-like projections on the proximal end of the ungual; separation of the flexor tubercle into two small, raised regions; proximal articular surface slightly pinched laterally (Fowler et al., 2020; Friemuth and Wilson, 2021). I also preliminarily refer two pedal unguals from the Lance Formation to †*Trierarchuncus prairiensis* based on a combination of features, including: paired lateral embayments on either side of the proximal end; slight curvature; absence of a flexor tubercle; a developed proximal lip and deepened nutrient grooves on either side of the ungual that suddenly direct ventrally at the proximal end (Salgado et al., 2009; Xu et al., 2013; Averianov and Sues, 2022). These unguals are identical in morphology to one another and are similar to the manual ungual in the development of the proximal lip and the lateral embayments.

Coelurosauria indet. taxon

*Material.—*Shed tooth crown recovered in same block as the holotype of YPM VP 59473 YPM VP 59473 (Figure S3).

*Locality and Horizon.—*Niobrara County, Wyoming, United States, North America. Recovered during the 1889 expedition lead by John Bell Hatcher.

*Comments.—*This ziphodont isolated tooth crown is assignable to a non-avian theropod dinosaur based on its curvature, mediolateral compression, and the presence of serrations along its distal carina. Although this tooth cannot be assigned to a family, it closely compares with teeth assigned to small-bodied tyrannosauroids and dromaeosaurids from the Late Cretaceous of the western interior (Larson and Currie, 2013; Williamson and Brusatte, 2014). This tooth is important for substantiating the best evidence that small-bodied, most probably feathered non-avian theropod dinosaurs coexisted with crown birds.

*Conclusion.-*In sum, the faunal survey resulted in the recognition of at least four distinct types of non-avian theropods, including small-bodied troodontids, eudromaeosaurs, small-bodied dromaeosaurids that probably belong within Microraptoria, and parvicursorine alvarezsaurs with strong similarities to †*Trierarchuncus prairiensis.* All of these are estimated to be considerably larger than the type specimen of YPM VP 59473 (e.g., Longrich and Currie, 2009a, 2009b; Longrich et al., 2011; Fowler et al., 2020). Combining the results of this faunal survey with that carried out by Longrich et al. (2011) shows that the Lance Formation theropod fauna is dominated by avian stem lineages.

**Table S1.** Measurements of selected elements of the holotype exposed or freed from matrix.

| Element | Measurements (mm) |
| --- | --- |
| Humerus | 17.65 (proximal max. diameter), 65.05 (part. proximodistal, est. 70), ~5.5 (shaft circumference) |
| Ulna | 32.81 (part. proximodistal) |
| Radius | 37.02 (part. proximodistal) |
| Tibiotarsus | 34.63 (proximodistal), 4.33 (max. shaft diameter) |
| Proximal pedal phalanx (III-1?) | 11.5 (proximodistal), 4.0 (distal depth) |
| Pedal ungual (IV-3?) | ~5.0 (proximodistal) |

**Table S2.** Biogeographic reconstruction model comparison.

| Model | AIC, AICc values |
| --- | --- |
| DEC | 292.60, 292.89 |
| DEC+j | 293.96, 294.58 |
| DIVALIKE | 298.31, 298.91 |
| DIVALIKE+j | 298.31, 298.91 |
| BAYAREALIKE | 291.01, 291.61 |
| BAYAREALIKE+j | 291.01, 291.61 |

*p-values from chi-squared tests for statistically significant differences in fit across models with and without the j parameter added were all >>0.05, indicating no discernable benefit to adding j.

**Table S3.** CT Scanning Parameters.

| scanned 1/6/2023 at YPM CT Scanning Facility | | |
| --- | --- | --- |
| Specimen | VP.059473 | VP.059473 |
| Taxon | bird | bird |
| Element | Left humerus; block (large) with radius, ulna, quadrate, skull fragments, femur fragment, ungual phalanx; block (small) with metatarsus, vertebrae, several pedal phalanges | Tibiotarsus, distal end of large phalanx, isolated theropod tooth, additional fragments including ungual phalanx |
| Requested by | Chase Brownstein | Chase Brownstein |
| Specimen location | 177.049Y, 438.785mag | 170.940Y, 617.990mag |
| Detector location | -180 | 0 |
| kV, uA | 104kV, 101uA | 90kV, 89uA |
| filter | no filter | no filter |
| file name | mf235_VP_59473_2 | mf235_VP_59473_1 |
| frames to average | 128 | 128 |
| images | 3 | 3 |
| projections | 3142 | 3142 |
| frames | 2 | 2 |
| Voxel size | 82.9264 μm | 81.0658 μm |

**IV. Supplementary References.**

- Averianov, A. and Sues, H.D., 2017. The oldest record of Alvarezsauridae (Dinosauria: Theropoda) in the Northern Hemisphere. *PLoS One*, *12*(10), p.e0186254.
- Averianov, A.O. and Sues, H.D., 2022. New material and diagnosis of a new taxon of alvarezsaurid (Dinosauria, Theropoda) from the Upper Cretaceous Bissekty Formation of Uzbekistan. *Journal of Vertebrate Paleontology*, *41*(5), p.e2036174.
- Benito, J., Kuo, P.C., Widrig, K.E., Jagt, J.W. and Field, D.J., 2022. Cretaceous ornithurine supports a neognathous crown bird ancestor. *Nature*, *612*(7938), pp.100-105.
- Bonaparte, J.F. (1991) Los vertebrados fósiles de la Formación Río Colorado, de la Ciudad de Neuquén y cercanías, Cretácico Superior, Argentina. Revista del Museo Argentino de Ciencias Naturales, 4, 17–123
- Cau, A. and Madzia, D., 2018. Redescription and affinities of *Hulsanpes perlei* (Dinosauria, Theropoda) from the Upper Cretaceous of Mongolia. *PeerJ*, *6*, p.e4868.
- Cau, A., Beyrand, V., Voeten, D.F., Fernandez, V., Tafforeau, P., Stein, K., Barsbold, R., Tsogtbaatar, K., Currie, P.J. and Godefroit, P., 2017. Synchrotron scanning reveals amphibious ecomorphology in a new clade of bird-like dinosaurs. *Nature*, *552*(7685), pp.395-399.
- Chiappe, L.M., Norell, M.A. and Clark, J.M., 2002. The Cretaceous, short-armed Alvarezsauridae: Mononykus and its kin. *Mesozoic birds: above the heads of dinosaurs*, pp.87-120.
- Currie, P.J., 1987. Bird-like characteristics of the jaws and teeth of troodontid theropods (Dinosauria, Saurischia). *Journal of Vertebrate Paleontology*, *7*(1), pp.72-81.
- Faux, C.M. and Padian, K., 2007. The opisthotonic posture of vertebrate skeletons: postmortem contraction or death throes?. *Paleobiology*, *33*(2), pp.201-226.
- Field, D.J., Benito, J., Chen, A., Jagt, J.W. and Ksepka, D.T., 2020. Late Cretaceous neornithine from Europe illuminates the origins of crown birds. *Nature*, *579*(7799), pp.397-401.
- Forster, C.A., O’connor, P.M., Chiappe, L.M. and Turner, A.H., 2020. The osteology of the Late Cretaceous paravian *Rahonavis ostromi* from Madagascar. *Palaeontologia Electronica*, *23*(2), pp.1-75.
- Fowler, D. W., Freedman, E. A., Scannella, J. B., & Kambic, R. E. (2011). The predatory ecology of Deinonychus and the origin of flapping in birds. *PLoS One*, *6*(12), e28964.
- Fowler, D.W., Wilson, J.P., Fowler, E.A.F., Noto, C.R., Anduza, D. and Horner, J.R., 2020. *Trierarchuncus prairiensis* gen. et sp. nov., the last alvarezsaurid: Hell Creek Formation (uppermost Maastrichtian), Montana. *Cretaceous Research*, *116*, p.104560.
- Freimuth, W.J. and Wilson, J.P., 2021. New manual unguals *of Trierarchuncus prairiensis* from the Hell Creek Formation, Montana, and the ontogenetic development of the functional alvarezsaurid hand claw. *Cretaceous Research*, *119*, p.104698.
- Gauthier, J., 1986. Saurischian monophyly and the origin of birds. *Memoirs of the California Academy of sciences*, *8*, pp.1-55.
- Gianechini, F.A., Makovicky, P.J., Apesteguía, S. and Cerda, I., 2018. Postcranial skeletal anatomy of the holotype and referred specimens of *Buitreraptor gonzalezorum* Makovicky, Apesteguía and Agnolín 2005 (Theropoda, Dromaeosauridae), from the Late Cretaceous of Patagonia. *PeerJ*, *6*, p.e4558.
- Gilmore, C. W. 1924. On *Troodon validus*, an ornithopodus dinosaur from the Belly River Cretaceous of Alberta, Canada. Bulletin of the Department of Geology. *University of Alberta.*, 1: 1–143.
- Hendrickx, C., Mateus, O., Araújo, R. and Choiniere, J., 2019. The distribution of dental features in non-avian theropod dinosaurs: Taxonomic potential, degree of homoplasy, and major evolutionary trends. *Palaeontologia Electronica*, *22*(3), pp.1-110.
- Hutchinson, J.R. and Chiappe, L.M., 1998. The first known alvarezsaurid (Theropoda: Aves) from North America. *Journal of Vertebrate Paleontology*, *18*(3), pp.447-450.
- Hwang, S.H., NORELL, M.A., Qiang, J.I. and Keqin, G.A.O., 2002. New specimens of *Microraptor zhaoianus* (Theropoda: Dromaeosauridae) from northeastern China. *American Museum Novitates*, *2002*(3381), pp.1-44.
- Keutgen, N., 2018. A bioclast-based astronomical timescale for the Maastrichtian in the type area (southeast Netherlands, northeast Belgium) and stratigraphic implications: the legacy of PJ Felder. *Netherlands Journal of Geosciences*, *97*(4), pp.229-260.
- Larson, D.W. and Currie, P.J., 2013. Multivariate analyses of small theropod dinosaur teeth and implications for paleoecological turnover through time. *PLoS One*, *8*(1), p.e54329.
- Larson, D.W., Brown, C.M. and Evans, D.C., 2016. Dental disparity and ecological stability in bird-like dinosaurs prior to the end-Cretaceous mass extinction. *Current Biology*, *26*(10), pp.132
- Lee, S., Lee, Y.N., Currie, P.J., Sissons, R., Park, J.Y., Kim, S.H., Barsbold, R. and Tsogtbaatar, K., 2022. A non-avian dinosaur with a streamlined body exhibits potential adaptations for swimming. *Communications Biology*, *5*(1), p.1185.
- Longrich, N.R. and Currie, P.J., 2009a. A microraptorine (Dinosauria–Dromaeosauridae) from the late Cretaceous of North America. *Proceedings of the National Academy of Sciences*, *106*(13), pp.5002-5007.
- Longrich, N.R. and Currie, P.J., 2009b. *Albertonykus borealis*, a new alvarezsaur (Dinosauria: Theropoda) from the Early Maastrichtian of Alberta, Canada: implications for the systematics and ecology of the Alvarezsauridae. *Cretaceous Research*, *30*(1), pp.239-252.
- Lü, J. and Brusatte, S.L., 2015. A large, short-armed, winged dromaeosaurid (Dinosauria: Theropoda) from the Early Cretaceous of China and its implications for feather evolution. *Scientific Reports*, *5*(1), p.11775.
- Marsh, O.C., 1881. Principal characters of American Jurassic dinosaurs, part V. *American Journal of Science*, *3*(125), pp.417-423.
- Matthew, W.D. and Brown, B., 1922. Article VI. The family Deinodontidae, with notice of a new genus from the Cretaceous of Alberta. *Bull Am Mus Nat Hist*, *156*, pp.367-385.
- Norell, M., Makovicky, P. J., Akademi, M. S. U., & Mongolian-American Museum Paleontological Project. (1999). Important features of the dromaeosaurid skeleton. 2, Information from newly collected specimens of *Velociraptor mongoliensis*. American Museum novitates; no. 3282.
- Norell, M.A., Makovicky, P.J., Bever, G.S., Balanoff, A.M., Clark, J.M., Barsbold, R. and Rowe, T., 2009. A review of the Mongolian cretaceous dinosaur *Saurornithoides* (Troodontidae: Theropoda). *American Museum Novitates*, *2009*(3654), pp.1-63.
- Ostrom, J.H., 1969. Osteology of *Deinonychus antirrhopus*, an unusual theropod from the Lower Cretaceous of Montana. Bulletin of the Peabody Museum of Natural History 30:1-173.
- Pei, R., Li, Q., Meng, Q., Gao, K.Q. and Norell, M.A., 2014. A new specimen of *Microraptor* (Theropoda: Dromaeosauridae) from the Lower Cretaceous of western Liaoning, China. *American Museum Novitates*, *2014*(3821), pp.1-28.
- Pei, R., Norell, M.A., Barta, D.E., Bever, G.S., Pittman, M. and Xu, X., 2017. Osteology of a new late Cretaceous troodontid specimen from Ukhaa Tolgod, Ömnögovi Aimag, Mongolia. *American Museum Novitates*, *2017*(3889), pp.1-47.
- Pittman, M., Xu, X. and Stiegler, J.B., 2015. The taxonomy of a new parvicursorine alvarezsauroid specimen IVPP V20341 (Dinosauria: Theropoda) from the Upper Cretaceous Wulansuhai Formation of Bayan Mandahu, Inner Mongolia, China. *PeerJ*, *3*, p.e986.
- Poust, A.W., Gao, C., Varricchio, D.J., Wu, J. and Zhang, F., 2020. A new microraptorine theropod from the Jehol Biota and growth in early dromaeosaurids. *The Anatomical Record*, *303*(4), pp.963-987.
- Salgado, L., Coria, R.A., Arcucci, A.B. and Chiappe, L.M., 2009. Restos de Alvarezsauridae (Theropoda, Coelurosauria) en la Formación Alien (Campaniano-Maastrichtiano), en Salitral Ojo de Agua, Provincia de Río Negro, Argentina. *Andean geology*, *36*(1), pp.67-80.
- Senter, P., Barsbold, R., Britt, B.B. and Burnham, D.A., 2004. Systematics and evolution of Dromaeosauridae (Dinosauria, theropoda). *Bulletin of the Gunma Museum of Natural History*, *8*, pp.1-20.
- Stirling, E.C. and Zietz, A.H.C., 1913. *Fossil remains of Lake Callabonna*. Rigby.
- Sues, H.D., 1978. A new small theropod dinosaur from the Judith River Formation (Campanian) of Alberta Canada. *Zoological Journal of the Linnean Society*, *62*(4), pp.381-400.
- Turner, A.H., Makovicky, P.J. and Norell, M.A., 2012. A review of dromaeosaurid systematics and paravian phylogeny. *Bulletin of the American museum of natural history*, *2012*(371), pp.1-206.
- Turner, A.H., Montanari, S. and Norell, M.A., 2021. A new dromaeosaurid from the Late Cretaceous Khulsan locality of Mongolia. *American Museum Novitates*, *2020*(3965), pp.1-48.
- Turner, A.H., Pol, D. and Norell, M.A., 2011. Anatomy of *Mahakala omnogovae* (Theropoda: Dromaeosauridae), Tögrögiin Shiree, Mongolia. *American Museum Novitates*, *2011*(3722), pp.1-66.
- Walker, J.D., and Geissman, J.W., compilers, 2022, Geologic Time Scale v. 6.0: Geological Society of America.
- White, M.A., 2009. The subarctometatarsus: intermediate metatarsus architecture demonstrating the evolution of the arctometatarsus and advanced agility in theropod dinosaurs. *Alcheringa*, *33*(1), pp.1-21.
- Williamson, T.E. and Brusatte, S.L., 2014. Small theropod teeth from the Late Cretaceous of the San Juan Basin, northwestern New Mexico and their implications for understanding latest Cretaceous dinosaur evolution. *PLoS One*, *9*(4), p.e93190.
- Xu, X., Upchurch, P., Ma, Q., Pittman, M., Choiniere, J., Sullivan, C., Hone, D.W., Tan, Q., Tan, L., Xiao, D. and Han, F., 2013. Osteology of the Late Cretaceous alvarezsauroid *Linhenykus monodactylus* from China and comments on alvarezsauroid biogeography. *Acta Palaeontologica Polonica*, *58*(1), pp.25-46.

**Figure S1. Dromaeosaurids and troodontids of the Lance Formation*.*** Troodontid tooth in (a) ?labial and (b) ?lingual views, compared to (c) the jaw and (d) tooth of a †*Troodon*-like troodontid from the Judith River Formation (YPM VPPU 22445). Distal half of metatarsal III in (e) lateral, (f) medial, and (g) dorsal views. Left pedal ungual II-3 in (h) medial, (i) proximal, and (j) lateral views. Left pedal phalanx II-1 in (k) medial, (l) lateral, (m) proximal, (n) dorsal, and (o) ventral views. Distal caudal vertebra in (p) lateral, (q) dorsal, and (r) ventral views. Proximal end of pedal phalanx II-2 in (s) lateral view. Abbreviations: distd, distal denticles, mesc, mesial carina, mesd, mesial denticles, coll, collateral ligament pit, buttrs, buttressing surface for adjacent metatarsal, ging, ginglymoid distal end, adex, apex of distal dorsal expansion, ns, neural spine, vens, ventral sulcus.

**Figure S2. Alvarezsaurids of the Lance Formation*.*** ?Left manual ungual I in (a) lateral, (b) medial, and (c) ventral views. Partial pedal ungual in (d) lateral, (e) medial, and (f) ventral views. Complete pedal ungual in (g) lateral, (h) medial, and (i) ventral views. Abbreviations: ng, neurovascular groove, for, foramen on ventral surface, ventr, ventral midline ridge, vents, ventral paired sulcus.

**Figure S3. Non-avian theropod tooth recovered with YPM VP 59473 *.*** The serrated tooth of a small-bodied theropod dinosaur recovered from the same matrix as YPM VP 59473 shown in multiple views.
