# Supplementary figures and images for: A juvenile bird with possible crown-group affinities from a dinosaur-rich Cretaceous ecosystem in North America
Source: BMC Ecol Evol. 2024 Feb 9;24:20. doi: 10.1186/s12862-024-02210-9 (PMC10858573; doi:10.1186/s12862-024-02210-9)

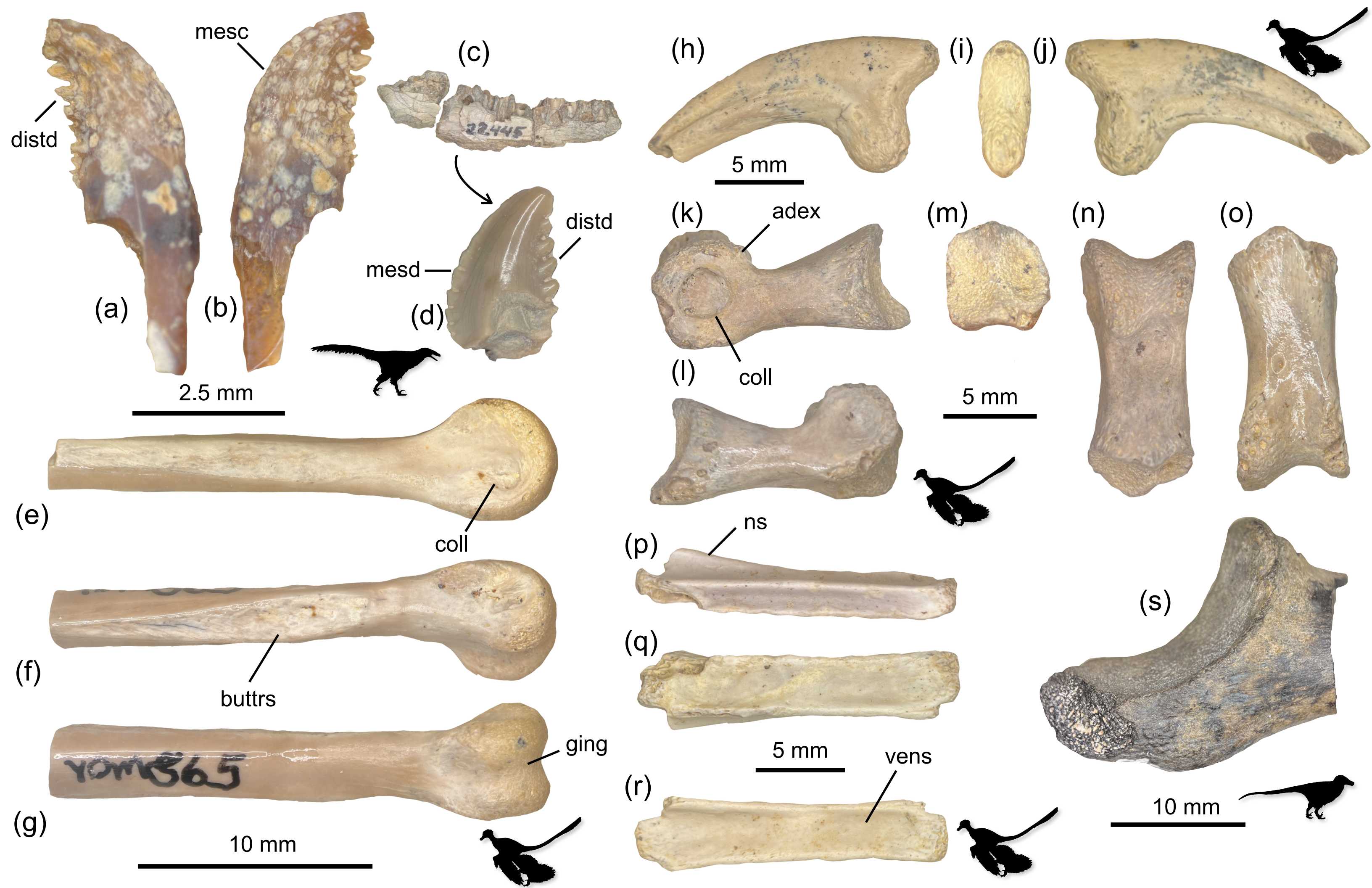

Supplement: Supplementary file 2 — Additional file 2. [file 12862_2024_2210_MOESM2_ESM.pdf]

(a)

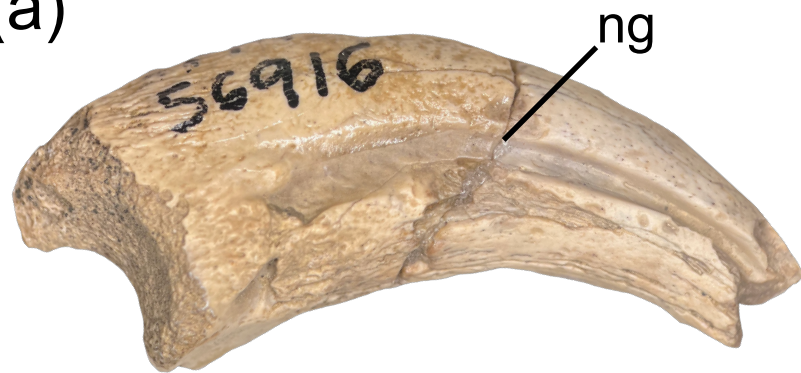

(b)

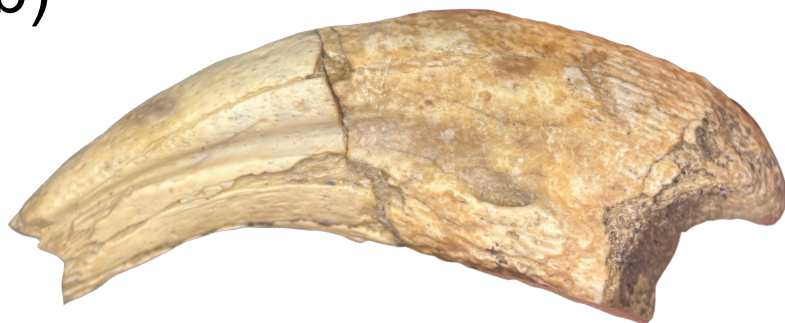

(c)

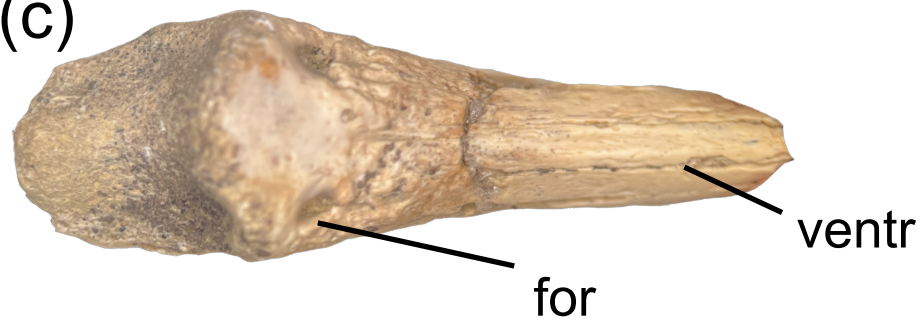

10 mm

(d)

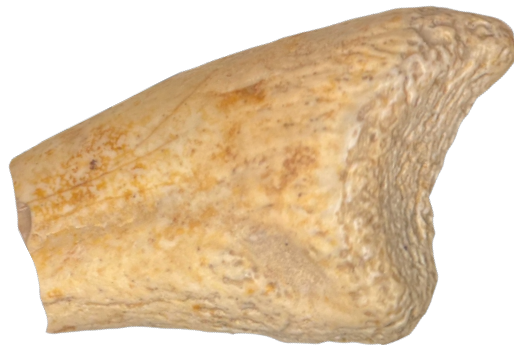

(e)

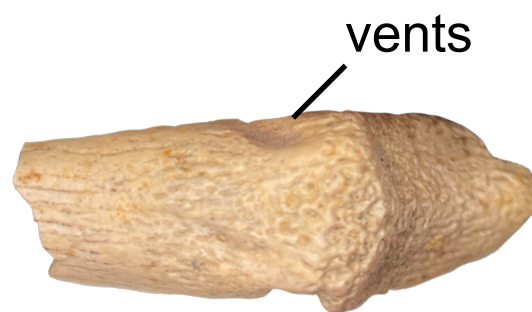

(f)

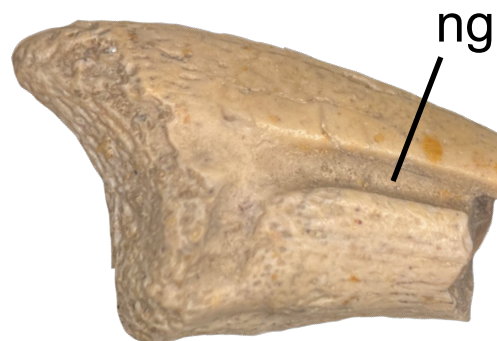

(g)

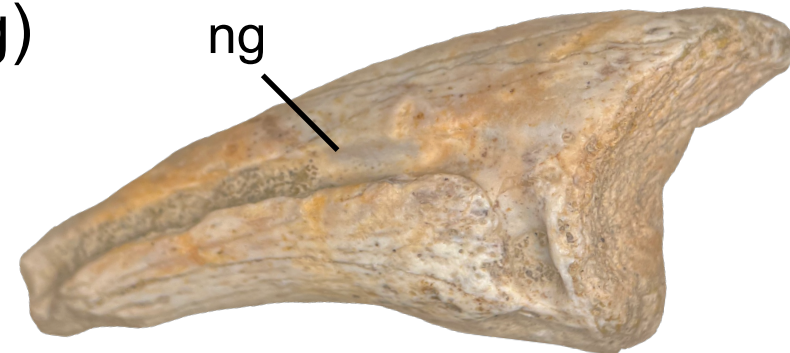

(h)

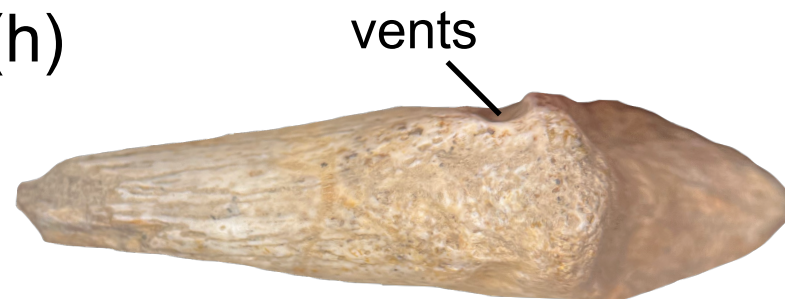

(i)

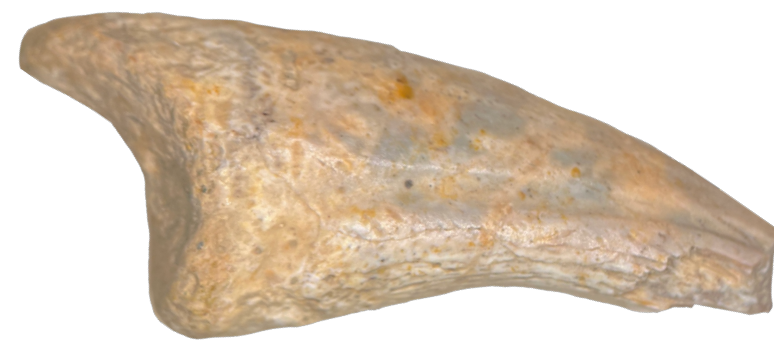

5 mm

Supplement: Supplementary file 3 — Additional file 3. [file 12862_2024_2210_MOESM3_ESM.pdf]

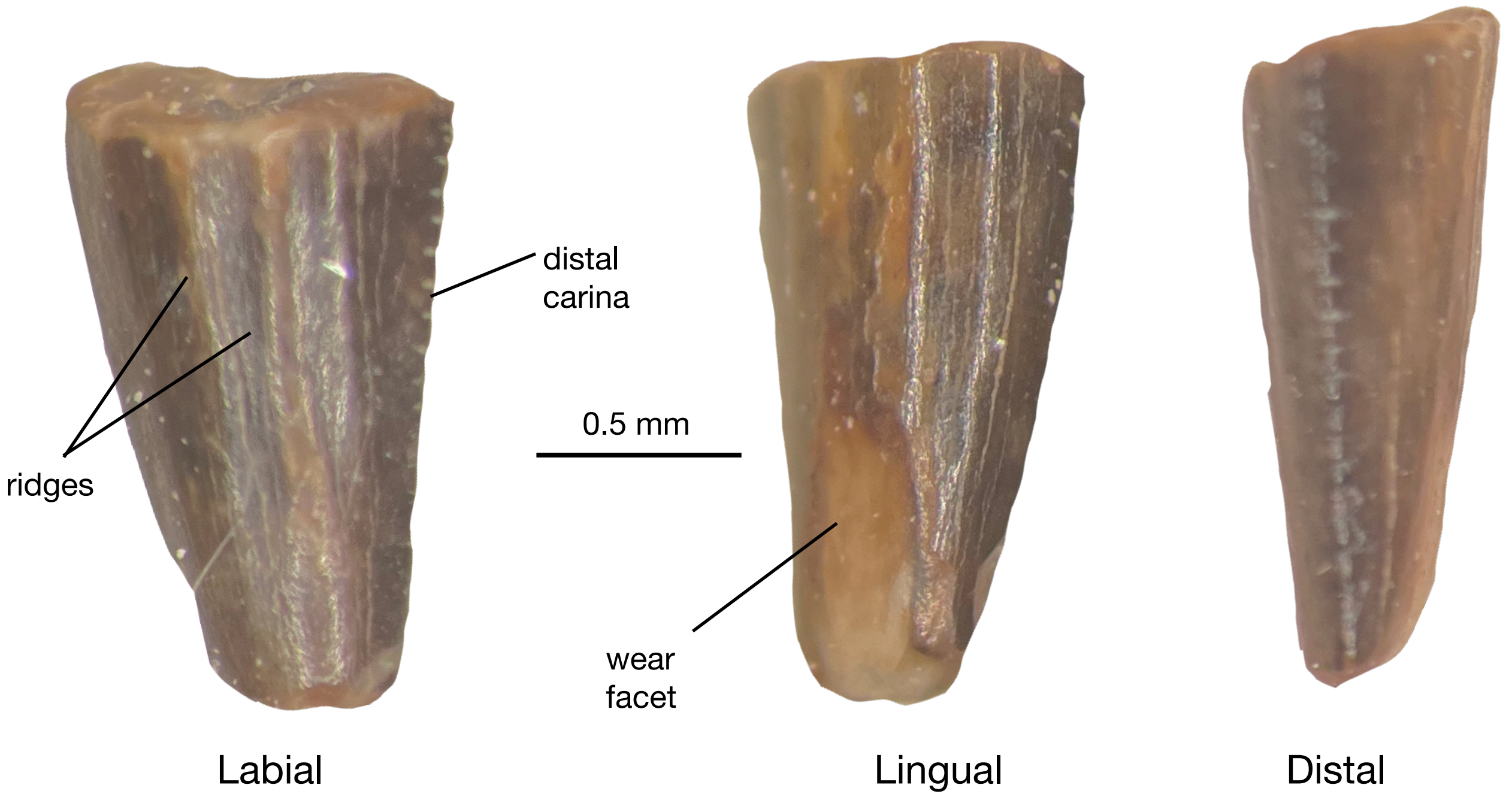

Supplement: Supplementary file 4 — Additional file 4. [file 12862_2024_2210_MOESM4_ESM.pdf]
